# Nrf2 Activation: Involvement in Central Nervous System Traumatic Injuries. A Promising Therapeutic Target of Natural Compounds

**DOI:** 10.3390/ijms24010199

**Published:** 2022-12-22

**Authors:** Serena Silvestro, Emanuela Mazzon

**Affiliations:** IRCCS Centro Neurolesi “Bonino-Pulejo”, Via Provinciale Palermo, Contrada Casazza, 98124 Messina, Italy

**Keywords:** Nrf2, oxidative stress, spinal cord injury, traumatic brain injury

## Abstract

Central nervous system (CNS) trauma, such as traumatic brain injury (TBI) and spinal cord injury (SCI), represents an increasingly important health burden in view of the preventability of most injuries and the complex and expensive medical care that they necessitate. These injuries are characterized by different signs of neurodegeneration, such as oxidative stress, mitochondrial dysfunction, and neuronal apoptosis. Cumulative evidence suggests that the transcriptional factor nuclear factor erythroid 2-related factor 2 (Nrf2) plays a crucial defensive role in regulating the antioxidant response. It has been demonstrated that several natural compounds are able to activate Nrf2, mediating its antioxidant response. Some of these compounds have been tested in experimental models of SCI and TBI, showing different neuroprotective properties. In this review, an overview of the preclinical studies that highlight the positive effects of natural bioactive compounds in SCI and TBI experimental models through the activation of the Nrf2 pathway has been provided. Interestingly, several natural compounds can activate Nrf2 through multiple pathways, inducing a strong antioxidant response against CNS trauma. Therefore, some of these compounds could represent promising therapeutic strategies for these pathological conditions.

## 1. Introduction

Injuries to the central nervous system (CNS), such as traumatic brain injury (TBI) and spinal cord injury (SCI), represent a serious medical burden. The heterogeneity of the lesions by age and severity makes it difficult to find appropriate treatments [1,2,3].

The primary lesion triggers a dramatic inflammatory response that results in the activation of microglia, the invasion of peripheral immune cells, and the release of inflammatory mediators that damage tissue and lead to scar formation [4]. In this way, over the hours or months following the insult, the secondary lesion induces necrosis and apoptosis in both the damaged and healthy peripheral tissue [5]. The pathophysiological mechanisms underlying CNS lesions are complex and involve an inflammatory response, oxidative stress, mitochondrial dysfunction, blood-brain barrier (BBB) disruption, and so on. Among these, oxidative stress, one of the important mechanisms, occurs at the beginning of the damage and accompanies the entire traumatic event [6]. The reactive oxygen species (ROS) are small molecules derived from oxygen that are highly responsive, such as peroxides, superoxides, hydroxyl radicals, and singlet oxygen. ROS can affect both the severity and size, as well as the progression of TBI and SCI [7]. Under physiological conditions, a certain ROS level is important to maintain normal cellular function and adequately balance ROS levels [8]. However, an imbalance of these molecules results in their harmful interaction with nucleic acids, carbohydrates, lipids, and proteins [9,10]. Indeed, the interaction of these molecules with the lipid double layer determines the lipid peroxidation that induces damage and cell death [11]. Furthermore, it has been demonstrated that after TBI and SCI, oxidative stress can induce the oxidation of mitochondrial proteins, with a consequent reduction of mitochondrial activity and bioenergetic capacities [12,13]. However, low levels of these modifications, under physiological conditions, can serve the cell as beneficial responses to stress [14,15]. Contrarily, high levels of these modifications are responsible for enzymatic dysfunctions. In this regard, in recent decades, nuclear factor erythroid 2-related factor 2 (Nrf2) has received more attention due to its important role in cellular defense against different endogenous and exogenous stresses [16,17].

Nrf2 is a pleiotropic transcription factor that is responsible for the defense mechanism against oxidative stress and inflammatory damage, by regulating the expression of several genes [16]. Cumulative evidence suggests that Nrf2 plays a crucial role in orchestrating the antioxidant response in the brain [18], promoting the expression of several antioxidant enzymes, such as heme oxygenase-1 (HO-1), NADPH Quinone Dehydrogenase 1 (NQO1), glutathione (GSH) peroxidase, and other antioxidant proteins [19]. Indeed, Nrf2 demonstrates a protective role in many pathological processes, and its activation is involved in several CNS diseases [18,20], including TBI and SCI [21,22].

In this review, the pathophysiology of SCI and TBI will be illustrated, describing in detail the potential role of Nrf2 underlying oxidative stress as a trigger in the pathological process of these conditions. The biological role of Nrf2 and its involvement in the regulation of the intracellular redox balance will also be mentioned. There is growing interest in understanding the molecular mechanisms underlying Nrf2 activation or inhibition, for potential therapeutic purposes. In this regard, this article intends to analyze in depth the mechanism of Nrf2 as the main regulator of oxidative stress, also summarizing the natural compounds based on the regulation of the Nrf2 pathway for the future development of more effective treatments for TBI and SCI.

## 2. Methodology

**Figure 1:**
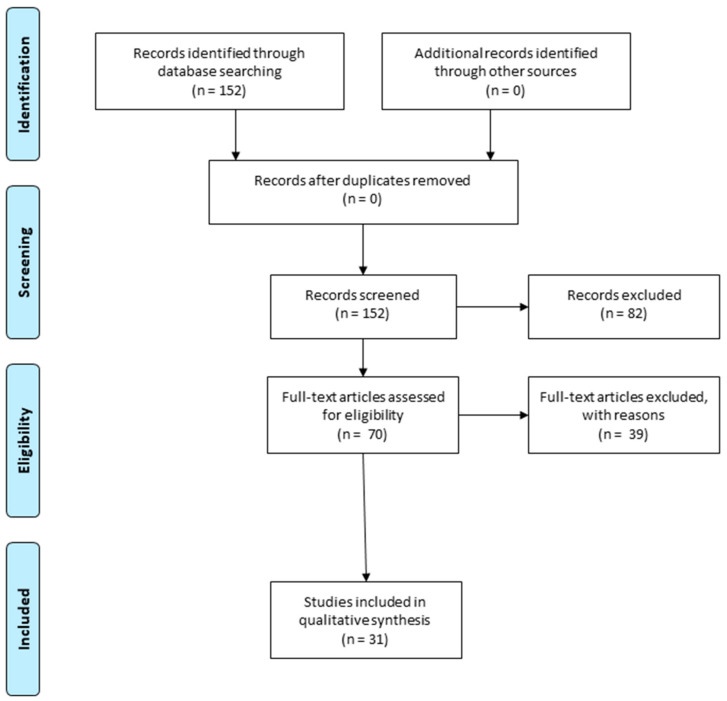
Prisma flow diagram details the methodology that was applied to choose the preclinical studies that were used for the writing of the review. Duplicate articles were excluded from the total number of studies that were recorded. Instead, we selected the articles that describe the role of natural compounds in the activation of Nuclear Factor Erythroid-2 Related Factor 2 (Nrf-2) signaling, which is important to promote injury repair and restore functional deficits (The PRISMA Statement is published in [23]).

In this review, the selected publications range from 2018 to 2022. In order to write the paragraphs, “5. Therapeutics interventions targeting the Nrf-2 in Spinal Cord Injury (SCI)” and “6. Therapeutics interventions targeting the Nrf-2 in Traumatic Brain Injury (TBI)”, the bibliography research in PubMed was performed using the following keywords: “spinal cord injury”, or “traumatic brain injury”, and “Nrf-2” and “oxidative stress”. We have specifically selected in vivo and in vitro studies that describe the progress of the investigations of natural compounds in the activation of Nrf2 signaling and their potential applications in TBI and SCI management (Figure 1).

## 3. The Biological Role of Nrf-2

**Figure 2:**
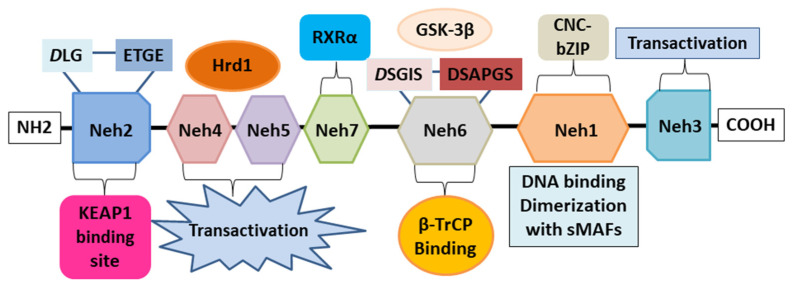
The structural architecture of Nrf2. The structure of Nrf2 contains seven domains, Neh domains, Neh1-Neh7. Neh1 contains a bZip motif, where the basic region is responsible for DNA binding and the zip dimerizes with other binding partners such as sMAFs. Neh2 contains ETGE and DLG motifs, involved in the interaction with Keap1 and subsequent Keap1-mediated proteasomal degradation. Neh3, Neh4, and Neh5 domains are transactivation domains of Nrf2. In particular, Neh4 and five domains interact with the Hrd1 responsible for Nrf2 degradation. The Neh6 domain contains two redox-independent degrons, DSGIS and DSAPGS, that bind to the E3 ubiquitin ligase β-TrCP involved in the Nrf2 degradation in oxidatively stressed cells. The neh7 domain mediates the interaction with RXRα, which represses Nrf2 activity. Nuclear factor erythroid 2-related factor 2: Nrf2; Nrf2-ECH homology: Neh; basic region leucine zipper: bZip; Cap’n’Collar: CNC; small muscleaponeurotic fibrosarcoma: sMAF; Kelch-like erythroid cell-derived protein with CNC homology-associated protein 1: Keap1; β-transducing repeat-containing protein: β-TrCP; retinoic X receptor alpha: RXRα; Glycogen synthase kinase-3: GSK-3β; HMG-CoA reductase degradation 1: Hrd1.

Nrf2, encoded in humans by the *NFE2L2* gene, is a transcription factor that regulates the gene expression of several antioxidant and cytoprotective enzymes through a promoter sequence known as the antioxidant response element (ARE). ARE is a promoter element found in many cytoprotective genes, and therefore, Nrf2 plays an important role in the cellular defense systems against ARE-dependent environmental stresses. Nrf2 belongs to the Cap’n’Collar (CNC) subfamily of basic region leucine zipper (bZIP) transcription factors [24]. Nrf2 possesses seven conserved Nrf2-ECH homology (Neh) domains with different functions to control Nrf2 transcriptional activity (Figure 2). The bZip in the Neh1 domain interacts with AREs for gene transcription activation, while the Neh2 domain specifically recognizes the domain of Kelch-like erythroid cell-derived protein with CNC homology-associated protein 1 (Keap1), to mediate the ubiquitination and degradation of Nrf2. The Neh3-5 domains bind various transcriptional components, acting as transcriptional activation domains, while the Neh6 domain binds the E3 ubiquitin ligase protein β-transducin repeat-containing protein (β-TrCP), involved in the degradation of Nrf2 in cells subjected to oxidative stress [25]. The Neh7 domain mediates interaction with the retinoic X receptor alpha (RXRα), a repressor of Nrf2 activity [26]. The presence of multiple domains allows Nrf2 to regulate the transcriptional activation of its target genes at different levels, thus regulating transcriptional, post-transcriptional, and post-translational regulation. Nrf2 activation is induced during oxidative stress, inflammation, and exposure to various stimuli such as growth factors, allowing cells to respond to different forms of stress. Indeed, Nrf2 is responsible for the regulation of more than 200 genes, all of which have the ARE promoter. Nrf2-regulated genes encode enzymes involved in xenobiotic and endobiotic metabolism, inflammatory responses, oxidative stress, and lipid, carbohydrate, and protein degradation metabolism [27].

Nrf2 is a transcription factor expressed in all cell types and is regulated, under different conditions, mainly by four ubiquitylation, by ubiquitin ligase E3, and by proteasomal degradation. Under physiological conditions, its basal protein levels are generally kept low due to proteasome degradation mediated by its negative regulator Keap1 [28]. Indeed in the cytoplasm, Keap1 creates a ubiquitin E3 ligase complex with Cullin3 which, by targeting Nrf2, induces its polyubiquitination and the rapid degradation of the proteasome [29,30]. During oxidative stress, electrophiles and ROS react with Keap1 sensor cysteines, protecting Nrf2 from Keap1-driven polyubiquitination and proteasome degradation [31,32]. This induces the translocation of the Nrf2 into the nucleus, where its accumulation allows the binding with the small muscleaponeurotic fibrosarcoma (sMAF) oncogene, with consequent activation of the expression of cytoprotective genes containing ARE [28,30,33]. Nrf2 activity is modulated at multiple levels, including regulation of the stability of the Nrf2 protein. Glycogen synthase kinase-3 β (GSK-3β) phosphorylates a motif present in the Neh6 domain, promoting the ubiquitination and degradation of Nrf2 [34,35]. GSK-3β phosphorylates Nrf2, creating a degradation domain that is recognized by β-TrCP and tagging for proteasomal degradation by the Keap1-Cullin 1 (CUL1) and RING-box protein 1 (RBX1) complex [36,37]. Likewise, CR6-interacting Factor 1, involved with proteins inducible by DNA damage, by binding the Neh2 domain and the C-terminal regions of Neh1 and Neh3 induce the degradation of Neh2 [38]. Nrf2 activity is also modulated by post-translational modifications that induce changes in its binding partners. Some proteins, such as c-jun N-terminal kinase (JNK) [39], protein kinase C (PKC) [40], extracellular signal-regulated protein kinases (ERK) [39], protein kinase R-like endoplasmic reticulum kinase [41], phosphoinositide 3-kinase (PI3K)/AKT, and casein kinase 2 [42], regulate Nrf2 phosphorylation by increasing its stability and transcriptional activity. Instead, proteins such as p38 and GSK-3β induce the phosphorylation of Nrf2 reducing its stability [35].

It is known that the Nrf2-Keap1 axis exerts a protective effect in various disorders that see oxidative stress and inflammation as the main pathological mechanisms [43]. Nrf2 is initially controlled at the transcriptional level by several transcription factors, such as the nuclear factor kappa-light chain-enhancer of activated B lymphocytes (NF-ĸB) [44,45]. The NFE2L2 promoter contains an NF-ĸB binding site which allows it to regulate Nrf2. Some signaling pathways, such as the PI3K-AKT pathway [46] and the Notch signaling pathway [47], seem to increase *NFE2L2* transcription in order to allow Nrf2 to carry out the basal Nrf2 antioxidant program. It was discovered that at the post-transcriptional level it can also be regulated by microRNAs such as miR-144, which appears to be linked to a reduction in the transcriptional Nrf2 activity [48]. Other miRNAs, such as miR-28 [49], miR-34 [50], miR-93 [51], and miR-153 [52], appear to be involved in the regulation of Nrf2.

**Figure 3:**
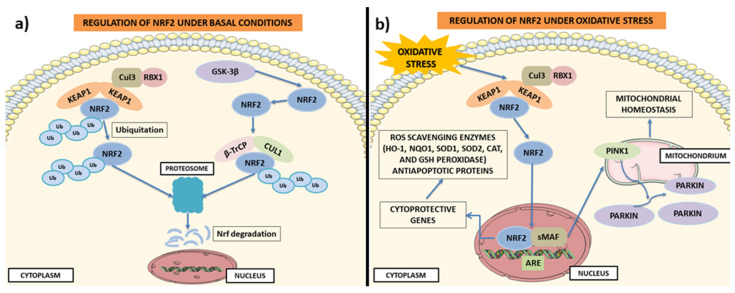
Regulation of the Nrf2 pathway. (**a**) Regulation of Nrf2 under basal conditions. Under basal conditions, Nrf2 is sequestered by Keap1 in the cytosol and ubiquitinated. The formation of the Keap1-Cul3-Rbx1 complex induces the degradation of the Nrf2 proteasome. In this way, Nrf2 expression is maintained in the cytosol at low levels. However, the degradation of Nrf2 can also be promoted by GSK-3β. GSK-3β by phosphorylating Nrf2 forms a recognition motif for the E3 ligase adapter β-TrCP. Formation of the GSK-3β/β-TrCP/Cul1 complex promotes Keap1-independent degradation of Nrf2. (**b**) Regulation of Nrf2 under oxidative stress. When cells are under severe oxidative stress, Nrf2 is released from Keap1 binding and is translocated from the cytosol to the nucleus. In the nucleus, it binds to ARE gene sequences and promotes the transcription of cytoprotective genes responsible for the translation of ROS scavenging enzymes (HO-1, NQO1, SOD, SOD2, CAT and GSH peroxidase) and antiapoptotic proteins. Furthermore, Nrf2 positively regulates PINK1 by promoting mitochondrial homeostasis through several mechanisms, such as the removal of damaged mitochondria. The image was created using the image bank of Servier Medical Art (Available online: http://smart.servier.com/, accessed on 15 November 2022), licensed under a Creative Commons Attribution 3.0 Unported License (Available online: https://creativecommons.org/licenses/by/3.0/, accessed on 15 November 2022). Nuclear factor E2-related factor 2: Nrf2; antioxidant response element: ARE; small muscleaponeurotic fibrosarcoma: sMAF; Kelch-like erythroid cell-derived protein with CNC homology-associated protein 1: Keap1; β-transducing repeat-containing protein: β-TrCP; Cullin 3: CUL3; RING-box protein 1: RBX1; PTEN Induced Kinase 1: PINK1; Glycogen synthase kinase-3 β: GSK-3β; heme oxygenase-1: HO-1; NADPH Quinone Dehydrogenase 1: NQO1; reactive oxygen species: ROS; superoxide dismutase: SOD; glutathione: GSH; catalase: CAT.

Nrf2/ARE signaling protects cells and tissues from oxidative stress [53] by negatively controlling the NF-kB signaling pathway that regulates inflammatory responses and cell damage [54]. Indeed, once translocated to the NF-κB nucleus, it induces the expression of proinflammatory cytokines (IL-1, IL-6, TNF-α), COX-2, and iNOS. Nrf2 inhibits the transcription of NF-κB by inhibiting its nuclear translocation [55]. Furthermore, high levels of Nrf2 enhance the levels of cellular HO-1, and consequently, increase the expression levels of phase II enzymes, blocking the degradation of IκB-α and the inhibitor of IκB-α [56]. Nrf2 can directly modulate ROS and RNS activating antioxidant enzymes, such as superoxide dismutase (SOD), GSH peroxidase, and members of peroxiredoxin [57,58]. Following oxidative stress, Nrf2 translocates into the nucleus where it induces the transcription of several antioxidant enzymes, such as GSH-dependent antioxidant enzymes (glutathione peroxidase 2, GPX2) and glutathione S-transferases (GST) [58]. Nrf2 also promotes the conversion of glutathione disulfide (GSSG) to GSH by glutathione reductase (GSR) [59], reducing oxidized thioredoxins by thioredoxin reductase (TrxR) [60] and Prx-SO2H by sulfiredoxin (Srx), as illustrated in Figure 3 [61].

Noteworthily, Nrf2 regulates mitochondrial function through the regulation of some key metabolic genes, for example, by improving glycolytic flux, amino acid metabolism, and glutaminolysis. Active Nrf2 is also involved in maintaining the integrity of mitochondrial DNA, an important regulator of cell death and inflammation [62]. Furthermore, Nrf2 is also a regulator of mitochondrial biogenesis via the activation of the mitochondrial transcription factor A (TFAM), the mitochondrial transcription factor B2 (TFBM2), and the nuclear respiratory factor-1 (Nrf-1) [63]. Additionally, regulating the expression of the transcriptional co-activator peroxisome proliferator activator gamma co-activator 1 alpha (PGC-1α), which is a major regulator of mitochondrial function and biogenesis, is another example of Nrf2 activity [64]. Additionally, when cells are subjected to oxidative stress, Nrf2 influences mitochondrial biogenesis also by inducing the process of mitophagy [65,66]. Nrf2 improves autophagy by promoting the expression of autophagic genes encoding SQSTM1/p62 [67], protein 2 containing the calcium-binding domain and spiral coil (CALCOCO2/NDP52), kinase 1 similar to unc-51 (ULK1), protein autophagic 5 (ATG5), and gamma-protein-like 1 associated with the aminobutyric acid receptor (GABARAPL1) [68].

For the purpose of improving the neuroprotective potential of Nrf2 signaling pathway activation, several natural flavanol compounds with antioxidant, anti-inflammatory, and iron-chelating properties have been tested. In this regard, it was demonstrated that intravenous or oral administration of epicatechin passes the BBB, improving vascular function and leading to the reduction both of oxidant species and free radicals through the activation of the Nrf2 signaling pathway [69]. Besides the activation of the Nrf2 pathway, it was highlighted that epicatechin can act also on activator protein-1 (AP-1) signaling pathways, thus protecting the astrocytes exposed to hemoglobin [70]. Additionally, it was demonstrated that epicatechin has a neuroprotective effect in the brain after TBI, by activating the Nrf2 pathway and inhibiting the expression of HO-1, and reducing iron deposition [71].

It is known that HO-1 overexpression could alter iron homeostasis and aggravate iron deposition in the brain. Excess iron is a pathological feature, promoting ROS generation and subsequent DNA damage; lipid peroxidation leads to BBB destruction, neuronal death, and neurological deficits [72,73]. Second, HO-1 overexpression and increased brain iron deposition could be linked to chronic inflammation. A previous study [74] demonstrated that exacerbated oxidative stress and iron accumulation could be promoted by inflammatory processes. Thus, natural compounds such as epicatechin, by activating Nrf2 and also inhibiting HO-1 expression and iron deposition, may show important neuroprotective effects. Nevertheless, several studies in Nrf2- and HO-1-deficient mice confirmed the neuroprotective function of Nrf2, and the deleterious effects of HO-1 in the pathologic process of intracerebral hemorrhage [20,75].

## 4. Pathophysiology of SCI

SCI is a pathological condition with a high mortality rate, and it reduces life expectancy depending on the severity of the insult and the age of the patient [76]. The available treatments for this condition are generally aimed at relieving the symptoms of patients by improving their quality of life [77]. Most spinal cord injuries are due to road accidents, falls, assaults, and sports injuries [78]. However, SCI can also be caused by other conditions, such as musculoskeletal diseases, congenital problems such as spinal bifida, infectious diseases, and cancers [79].

The initial phase immediately following the injury is known as the “primary injury”, it is an irreversible process that can be caused by bone fragmentation and ligament tear [80,81]. The primary lesion is followed by a cascade of events, including increased cell permeability, apoptotic signaling, excitotoxicity, and vascular and inflammatory damage, which will eventually lead to neuronal damage and death, known as the “secondary injury” [82,83,84]. The secondary lesion can be divided into three phases: acute (within a few days), subacute (from 2 days to 2 weeks), and chronic (over 2 weeks). During the acute phase, vascular damage can cause bleeding and disruption of the spinal cord barrier, which attracts the rapid infiltration of inflammatory cells such as neutrophils, resulting in the release of various pro-inflammatory cytokines, such as tumor necrosis factor-alpha (TNF-α), interleukin 1-alpha (IL-1α), interleukin 1-beta (IL-1β), and interleukin-6 (IL-6) [85,86]. During the subacute phase, arterial vessel damage impairs vascular supply and causes edema, leading to further neuronal and vascular damage, in particular causing the death of the oligodendrocytes responsible for the demyelination of the axons [87,88]. Furthermore, during this phase, the alteration of ionic homeostasis causes mitochondrial dysfunction [89,90], responsible for the excessive production of ROS and reactive nitrogen species (RNS) contributing to tissue damage [91]. During the chronic SCI phase, the damage expansion leads to the activation of astrocytes that produce excessive levels of the extracellular matrix. This alters vascular remodeling, the composition of the extracellular matrix, and the reorganization of neural circuits, inducing the formation of cystic cavities, axonal death, and the maturation of glial scars [92,93,94].

Since the complex pathophysiological changes make the secondary lesion more lethal than the primary one, the most promising approach in the treatment of SCI is to inhibit the secondary lesion and promote functional recovery [95]. In this regard, a large amount of evidence has shown that Nrf2 is involved in the pathogenesis of SCI and easily responds to traumatic injuries [96].

## 5. Therapeutics Interventions Targeting the Nrf-2 in SCI

Several evidences have shown that the Nrf2 improves spinal cord ischemia–reperfusion injury in neurons, and astrocytes, by activating antioxidant, antiapoptotic, and survival neuronal responses [97,98]. Therefore, strategies that can activate the Nrf2 pathway could be employed as a therapeutic tool to reduce oxidative stress and inhibit apoptotic processes that are activated in the damaged spinal cord. In a study performed on SCI rats, the effects of polyadine on oxidative stress, apoptosis, and the Nrf2 pathway underlying the SCI were evaluated. Polydatin administration (20 or 40 mg/kg, intraperitoneal) improved spinal cord impairment and locomotor function in SCI rats and also reduced injury-induced congestion, edema, and structural damage. Polydatin upregulated SOD and reduced malondialdehyde (MDA) levels, decreasing oxidative damage. Furthermore, it also decreased the levels of caspase-3 and split Bax and increased the levels of Bcl-2 in the spinal cord tissues, demonstrating its anti-apoptotic effect. Polydatin may have exerted its antioxidant and antiapoptotic action probably via the Nrf2 signaling pathway. Indeed, after treatment with the compound, the levels of nuclear Nrf2 and cytoplasmic HO-1 in spinal cord tissues were significantly elevated. Consistent with the findings observed in spinal cord tissues, including lipopolysaccharides (LPS)-stimulated BV2 microglia, polydatin treatment significantly reduced ROS and lactate dehydrogenase (LDH) production and inhibited BV2 cell apoptosis. These results, instead, were reversed with the Nrf2-knockdowns (KO) in LPS-induced BV2 cells [99].

Rosmarinic acid is a water-soluble polyphenolic phytochemical compound that has been shown to be protective against ischemic stroke [100], and against the neuronal cell damage induced by H_2_O_2_ [101]. These findings prompted researchers to investigate the effect of this compound on SCI and its underlying molecular mechanisms. After 7 days from the initial injury, the administration of rosmarinic acid (10, 20 or 40 mg/kg, intraperitoneal) reduced the areas of hemorrhage and swelling, the area of nerve cell destruction, reduced glial cell proliferation, and inflammatory cell infiltration. The treatment also increased the number of neurons in a dose-dependent manner, inducing functional recovery and a reduction in tissue damage, also after 28 days of treatment, highlighting the long-term effect of this compound. Like polydatin, rosmarinic acid also attenuated the damage-induced apoptosis by reducing the levels of Bax, cleaved caspase-9, and cleaved caspase-3, and increasing the level of Bcl-2. It promoted neuroprotection by increasing the expression of neurotrophic factors such as, neurofilament H (NF-H) and brain-derived neurotrophic factor (BDNF). Furthermore, the compound reduced oxidative stress by significantly increasing the activity of SOD, catalase (CAT), and GSH peroxidase, and reducing MDA. Subsequently, the proteomic analysis identified Nrf2 and NF-κB pathways as possible targets of rosmarinic acid, demonstrating that these beneficial effects could be mediated by this pathway. To confirm these data, the in vitro models of H_2_O_2_-induced oxidative injury and LPS-induced inflammatory injury in PC12 cells were used. PC12 cells is a cell line of rat, originated from the neural crest and widely used as model for neural differentiation as well as to evaluate the effects of differentiation. Additionally, in vitro, rosmarinic acid improved the antiapoptotic response and oxidative damage via Nrf2/HO-1 signaling [102].

Ginsenoside Rb1 is a natural compound that regulates the immune balance and acts as a scavenger of free radicals; therefore, the potential antioxidant effect has been evaluated in SCI. Treatment with ginsenoside Rb1 (10 mg/kg, intraperitoneal) improved hind limb motor function in rats that showed higher scores than those in the untreated SCI group. Histopathologically the compound reduced hemorrhage, neuronal degeneration/necrosis, and the infiltration of mononuclear cells and lymphocytes. After 7 days, the treatment significantly reduced serum MDA levels but increased SOD, CAT, and GSH levels. Instead, compared to the untreated SCI group, ginsenoside Rb1 significantly increased the expression of eNOS, HSP90, Nrf2, NQO1, and HO-1 proteins in the spinal cord. Ultimately, the SCI rats treated with ginsenoside Rb1 were injected with the eNOS inhibitor L-name to investigate the mechanism behind the protective effect. In this way, it was shown that ginsenoside Rb1 protected the spinal cord from damage-induced oxidative stress via the eNOS/Nrf2/ARE signaling pathway [103]. In another study, ginsenoside Rg1 decreased neuronal edema and bleeding in the damaged spinal cord and reduced inflammatory cell infiltration and cell necrosis. In addition, the treatment preserved the spinal cord structure from further injury and improved the motor function of the hind limbs in the SCI rat model. The treatment of SCI rats with ginsenoside Rb1 and Nrf2 inhibitor all-trans retinoic acid confirmed that the compound’s antioxidant and anti-inflammatory effects are regulated by the Nrf2/HO-1 signaling pathway [104]. The same results were obtained in rats administrated with notoginsenoside R1 (25 mg/kg/day, intraperitoneal) for 21 days, 2 h after SCI. These antioxidative, antiapoptotic, and anti-inflammatory effects of notoginsenoside R1 were reversed when SCI rats that had received notoginsenoside R1 were also treated with ML385, an inhibitor of the Nrf2/HO-1 signaling pathway. This confirmed that activation of the Nrf2/HO-1 signaling pathway after SCI may have an important implication in the management of SCI [105].

Luteolin, a flavonoid that possesses antioxidant [106], anti-inflammatory, and neuroprotective properties [107], has also been observed to be capable of activating Nrf2 gene expression. In in an ischemia–reperfusion SCI model, luteolin pretreatment (50 and 100 mg/kg, intraperitoneal) exhibited an improvement in neurological dysfunction of the lower hind limbs, demonstrating that this compound could protect animals from SCI. Pretreatment significantly reduced cellular apoptosis in the spinal cord of rats with an injury in a dose-dependent manner, and counteracted oxidative stress by lowering the oxidative activity of MDA and xantina oxidase. It also increased the antioxidant activity of SOD and GSH peroxidase and increased the levels of Nrf2 protein in the spinal cord 48 h after injury [108]. In this regard, in a subsequent study, it was explored whether pretreatment with this compound would improve recovery from SCI by activating Nrf2 and downstream target genes. Therefore, luteolin-pretreated animals were also treated with an Nrf2 inhibitor ML385 (30 mg/kg daily) for 14 consecutive days prior to the injury. Pretreatment with ML385 also reversed luteolin-induced pathological and functional improvements. Therefore, the results of the study showed that the anti-inflammatory, antioxidant, and neuroprotective activities of luteolin were closely related to the activation of Nrf2 [109].

Mulberrin is a natural compound of *Ramulus mori* which exhibits anti-inflammatory and antioxidant effects, therefore, it could be considered an effective therapeutic strategy to prevent the progression of SCI [110]. Mulberrin (15 or 30 mg/kg, by gavage), for 28 days, induced an improvement in motor function and attenuated the damage-induced apoptosis by the reduction of pro-apoptotic molecules (Bax, caspase-3, and PARP), and by the increase in the anti-apoptotic ones. Furthermore, the treatment resulted in a significant reduction of HO-1 and Nrf2 in the spinal cord tissue samples. These antioxidant effects of mulberrin were further confirmed in astrocytes treated with LPS in vitro. Therefore, the data of this study suggest that mulberrin could attenuate SCI by reducing miR-337 expressions which by regulating Nrf2 would reduce apoptosis, inflammation, and oxidative stress [111].

Another substance that appears to activate the Nrf2/HO-1 signaling pathway in the brain by attenuating oxidative stress and apoptosis-mediated cell death is maltol [112,113]. In the advanced phase of the damage (on the 7th and 14th days), the maltol (100 mg/kg, by gavage) improved locomotor function by decreasing the expression levels of the proapoptotic protein Bax and upregulating the antiapoptotic protein Bcl-2, thus attenuating neuronal apoptosis. Maltol could significantly inhibit H_2_O_2_-induced apoptosis in PC12 cells and could facilitate the expression of Nrf2 in the nucleus, thus suggesting that maltol could activate significantly the Nrf2 signaling pathway. This hypothesis was confirmed by the use of its ML385 inhibitor which reversed the inhibitory effect of maltol on ROS formation. Furthermore, maltol increased the level of mitophagy by activating the Nrf2/PINK1/Parkin signaling pathway in PC12 cells [114].

Perillaldehyde has been shown to be an activator of Nrf2 [115], therefore, it could be a potential therapeutic strategy for SCI. This hypothesis prompted a research group to explore these effects in an ischemia_—_reperfusion SCI rat model. The rats were pretreated for 7 days with perillaldehyde (36 and 72 mg/kg) administered intragastrically. Pretreatment-induced recovery of motor and neurological function also improved histological damage. In treated animals, functional and histological recovery was probably induced by the reduction of pro-inflammatory cytokines (IL-18 and IL-1β), by the inhibition of NLRP3 inflammasome activation, by the reduction of MDA, and by the increase in antioxidant enzymes (SOD, mitochondrial SOD, GSH peroxidase, and CAT). Furthermore, compared to the control group, the animals pretreated with the compound showed high Nrf2 and HO-1 expression. Moreover, pretreatment effectively inhibited the expression of phospho-NF-κB p65. The in vitro results also confirmed that perillaldehyde (0, 0.01, 0.1, 1, 10, 50, 100, 500 μM) counteracts the oxidative stress induced by the lesion probably by activating Nrf2/HO-1 [116]. Another natural compound that has been investigated for its possible role as an activator of Nrf2 is sinomenine [117,118]. After 14 days from the lesion, sinomenine (40 mg/kg, intraperitoneal) improved neurological deficits and reduced neuronal apoptosis, significantly decreasing inflammatory cytokines (IL-1β, IL-6, and TNF-α) and oxidative stress factors (MDA) in SCI rats. Contrarily, the treatment increased the activity of antioxidant enzymes such as SOD in the spinal cord. Furthermore, treatment with sinomenine induced an increase in the translocation of *Nrf2* from the cytoplasm to the nucleus. To evaluate the role of sinomenineas as an activator of Nrf2, H_2_O_2_-stimulated and LPS-stimulated PC12 cells were treated with Nrf2 small RNA interference, demonstrating that Nrf2 silencing blocks the antioxidant and anti-inflammatory effects of sinomenine (10 µM) [119].

**Figure 4:**
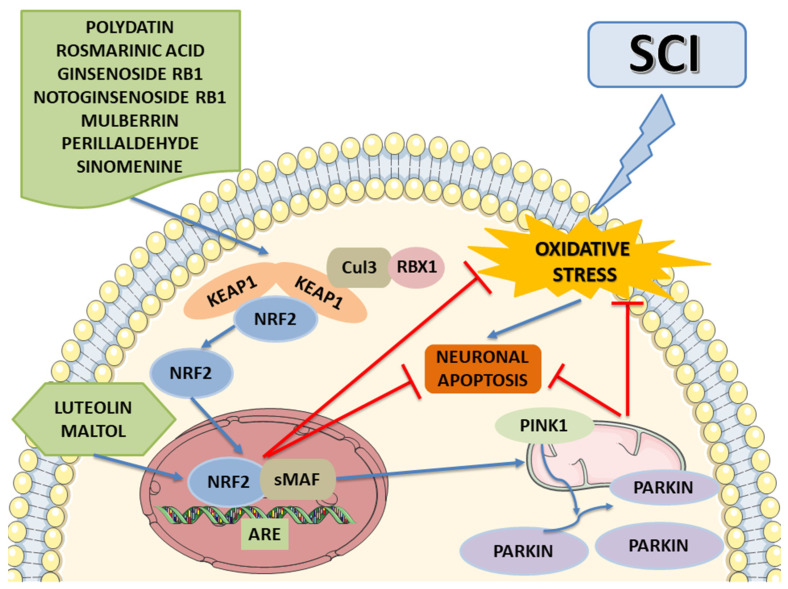
The potential molecular mechanism of natural compounds on SCI. These natural compounds can activate the expression of Nrf2 and facilitate Nrf2′s translocation from the cytosol to the nuclear, thus inhibiting oxidative stress and protecting from neuronal apoptosis. Moreover, some of these promote mitophagy via enhancing the Nrf2/PINK1/Parkin pathway, suppressing neuronal apoptosis in SCI. The image was created using the image bank of Servier Medical Art (Available online: http://smart.servier.com/, accessed on 15 November 2022), licensed under a Creative Commons Attribution 3.0 Unported License (Available online: https://creativecommons.org/licenses/by/3.0/, accessed on 15 November 2022). Spinal cord injury: SCI; nuclear factor E2-related factor 2: Nrf2; antioxidant response element: ARE; small muscleaponeurotic fibrosarcoma: sMAF; Kelch-like erythroid cell-derived protein with CNC homology-associated protein 1: Keap1; Cullin 3: CUL3; RING-box protein 1: RBX1; PTEN Induced Kinase 1: PINK1.

The data are summarized in Table 1, which shows that most natural compounds have a potential therapeutic efficacy for SCI management by inhibiting oxidative stress via Nrf2 activation (Figure 4). Indeed, once active, Nrf2 can downregulate inflammatory mediators (such as chemokines, and cytokines), and can downregulate NF-κB activity to achieve anti-inflammatory effects and regulate genes related to antioxidant mechanisms. Prevailing studies focus on the neuroprotective potential of these compounds by employing several mechanisms, including Nrf2/Keap1/ARE, Nrf2/PINK1/Parkin, NF-κB/TNF-α/ILs, and Bax/Bcl-2/caspases. Thus, the employment of these compounds that activate Nrf2 to effectively treat SCI could be a direction of future research.

## 6. Pathophysiology of TBI

TBI is defined as acquired intracranial damage caused by a mechanical injury that occurs in the brain and compromises its function. TBI is caused by an external force such as a bump, a jolt to the head, a severe blow from an object, or a deep puncture of the skull through brain tissue [120]. When patients maintain good neurological function and the sequelae can be resolved completely without any treatment, TBI is referred to as mild. Conversely, a TBI is defined as moderate or severe when the injury causes a disability that makes the quality of life negligible [121].

The pathophysiology of TBI is divided into primary and secondary lesions. The primary damage occurs at the time of the exposure to external force and causes a mechanical breakdown of brain tissue [122]. The secondary injury has its onset hours after the traumatic event and it is due to the cascade of biochemical, cellular, and physiological events that are activated after the injury responsible for further damages [123,124]. Within 24 h of TBI, there is a disruption of normal blood flow, which generally causes ischemic events responsible for increases in ionic gradients and oxidative phosphorylation with consequent accumulation of lactate. High concentrations of lactate cause neuronal damage and disruption of the BBB and cerebral edema [125]. These mechanisms cause the depolarization of neurons and an increase in neurotransmitters, such as glutamate and aspartate, responsible for calcium (Ca^2+^), potassium, and sodium homeostasis [126]. The increase in intracellular Ca^2+^ promotes the activation of enzymes, such as caspases, responsible for cell death by apoptosis.

Oxidative stress also plays an important role in the development and pathogenesis of TBI. Indeed, it has been shown that ROS production induces lipid peroxidation of cell membranes, especially at the axonal level, increasing the neurodegeneration process [126,127]. Soon after, these events also cause the activation of an inflammatory response that stimulates macrophages, glial T cell lymphocytes, and neutrophils, to release pro-inflammatory cytokines, such as TNFα, IL-1β, and IL-6, and also contribute to the production of superoxide radicals [128,129]. It is worth noting that an increase in iron is also generated, which catalyzes the reaction of oxygen radicals and induces ferroptosis [130]. In this regard, it has been shown that Nrf2 plays a protective role in TBI by counteracting the oxidative and inflammatory response [44].

## 7. Therapeutics Interventions Targeting the Nrf-2 in TBI

To date, there is no efficacy treatment against TBI; therefore, it is important to find effective therapeutic strategies for TBI treatment [131]. Same as for SCI, recent evidence has demonstrated that the activation of Nrf2 mediated by different natural compounds and drugs could be a valid therapeutic strategy for TBI.

Oridonine is the main constituent of *Rabdosia rubescens,* which acquired great interest for its role as a powerful activator of Nrf2 [132]. These properties prompted a research group to evaluate the potential effect of oridonine as an activator of Nrf2 in TBI management. Already 7 days after the injury, oridonine (20 mg/kg, intraperitoneal) improved the motor and cognitive function, reducing the volume of the cortical lesion, cerebral edema, the accumulation of macrophages reactivated after the lesion, and neuronal apoptosis. These outcomes are directly linked to the protective effect of oridonine against oxidative stress. Indeed, in the injured cortex a decrease in ROS, MDA, mitochondrial membrane, and adenosine triphosphate content was detected, which on the contrary were elevated after TBI. The same antioxidant effect of oridonine (0.5, 1, 2, and 4 μM) was also confirmed in H_2_O_2_-exposed mouse neuroblastoma (N2a) cells [133].

Breviscapine is an aglycone flavonoid extracted from the *Erigeron* plant that appears to be involved in the activation of ATPase and SOD after trauma [134]. Breviscapine (50 mg/kg, intraperitoneal) promoted the recovery of neurobehavioral functions and reduced lesion-induced apoptosis of neuronal cells. Indeed, in the brain tissues of treated TBI rats, the expression of Bax was inhibited, while that of Bcl-2 was increased. Furthermore, the treatment promoted the increased expression of Nrf2 and its related downstream proteins (HO-1 and NQO-1), consistent with mRNA levels, thus, highlighting its neuroprotective role via modulating Nrf2 [135].

Isoliquiritigenins are chalcone compounds, already known as a regulator of the Nrf2/ARE signaling pathway in animal models [136]. In line with these findings, isoliquiritigenin (20 mg/kg; intraperitoneally) one hour after injury improved neurological deficits, histopathologic lesion air, brain content, and vascular permeability. In agreement with the reduced water content, an increased expression of aquaporins was detected. Conversely, the expression level of the cleaved-caspase-3 was reduced, demonstrating the antiapoptotic effects exerted by the compound. The treatment counteracts TBI-induced oxidative stress by reducing the levels of MDA and increasing those of SOD and GSH peroxidase in the brain cortex tissue via Nrf2 activation. Indeed, in *Nrf2-KO* mice, isoliquiritigenin treatment failed to protect against damage-induced oxidative stress. These results obtained in vivo were also replicated in vitro in the OGD/R model using SH-SY5Y cells, where treatment with isoliquiritigenin (2, 5, 10, 20, and 40 µM) highlighted its role in activating the Nrf2 pathway, promoting the transfer of the Nrf2 protein from the cytoplasm to the nucleus [137].

7-D-Glucuronic acid-5,6-dihydroxyflavone (baicalin) is a flavonoid that crosses the BBB exerting neuroprotective effects in SCI [138]. These data have encouraged the study of the effects of this compound in a TBI mouse model. Baicalin (50, 100, and 150 mg/kg, intraperitoneal) improved neurological damage, reduced edema, and neuronal apoptosis, as demonstrated by an increased Bcl-2 protein expression and a decreased Bax and cleaved caspase-3. Furthermore, this compound exerted antioxidant effects by reducing MDA and increasing GSH peroxidase and SOD in the injured cerebral cortex. These effects are mediated by Nrf2 pathway activation, as demonstrated by the increased nuclear translocation of Nrf2 and its downstream antioxidant enzymes (NQO-1 and HO-1) after treatment with baicalin. Furthermore, the use of the PI3K/Akt inhibitor demonstrated the involvement of Akt in promoting the translocation of Nrf2 from the cytoplasm to the nucleus [139]. Another flavonoid that exerts antioxidant effects by activating Nrf2/HO-1 signaling in a PI3K/Akt-dependent manner is wogonin. Wogonin (40 mg/kg intraperitoneal), reduced cerebral edema, and improved behavioral deficits. This compound protected neuronal cells against apoptosis by increasing Bcl-2 protein expression in the CA1 region of the hippocampus of TBI rats and decreasing the apoptotic proteins caspase-3 and Bax. Furthermore, the compound exerted antioxidant actions, increasing the levels of antioxidant enzymes (GSH, CAT, and SOD) and decreasing ROS, MDA, and NOX2. Wogonin promoted the expression of phosphorylated Akt, Nrf2, and HO-1 in the hippocampus of TBI rats, demonstrating the antioxidant effects as mediated by the activation of Nrf2/HO-1 pathway in a PI3K/Akt-dependent manner [140]. One more flavonoid known for its antioxidant and neuroprotective effects is quercetin. In a model of TBI induced by a modified weight-drop device, treatment with intraperitoneal quercetin (5, 20, or 50 mg/kg) administered 0.5, 12, and 24 h after the injury, reduced cerebral edema and microgliosis 3 days after the injury. Furthermore, the compound counteracted oxidative stress by reducing MDA levels and increasing SOD, CAT, and GSH peroxidase in the cortex through Nrf2/HO-1 pathway activation [141]. The same result was also obtained following treatment with fisetin, another flavonoid extracted from vegetables and fruits [142], known for its anti-inflammatory and antioxidant properties [143]. Furthermore, after 3 days from the injury, treatment with fisetin (25, 50, and 75 mg/kg) protected against neuronal apoptosis, increasing Bcl-2 expression and reducing caspase-3 and Bax expression, and promoting the translocation of Nrf2 from the cytoplasm to the nucleus, thus activating the Nfr2/ARE pathway. Data was also confirmed by the use of *Nrf2-KO* mice, where the fisetin failed to counteract oxidative stress [144].

Another natural compound that exerts neuroprotective effects after TBI through possible involvement of the Nrf2-ARE pathway is curcumin, extracted from *Curcuma longa* L. Curcumin (50 and 100 mg/kg; intraperitoneal), especially at the higher dose, reduced injury-induced secondary brain damage, attenuated oxidative stress, and improved neurological function. Furthermore, the compound exerted anti-apoptotic effects by increasing the expression of Bcl-2 and reducing that of the cleaved caspase-3 in brain tissues. Furthermore, curcumin enhanced the translocation of Nrf2 from the cytoplasm to the nucleus, and by increasing the expression of downstream factors such as HO-1 and NQO1, demonstrated the role of this compound in counteracting the oxidative stress induced by the lesion [145]. In another study, at 24 h after the damage, curcumin reduced the levels of *Nrf2* and related proteins. In *Nrf2-KO* mice, the neuroprotective effects of curcumin treatment were significantly reduced, highlighting that the therapeutic effect of this compound in TBI is related to the activation of the Nrf2 pathway [146].

Sodium aescinate, extracted from chestnut seeds, showed anti-inflammatory properties and exerted antioxidant effects in an SCI model [147]. In this regard, a research group led by Zhang L. et al. explored the effects of this saponin in the TBI mouse model. Sodium aescinate (0.5, 1, and 2 mg/kg; intraperitoneal) reduced TBI-induced neurological deficits, brain injury, and counteracted oxidative stress and neuronal apoptosis. Treatment promoted *Nrf2* nuclear translocation and enhanced *Nrf2-ARE* binding. The same data were also obtained in vitro, suggesting that this compound provided neuroprotective effects, especially at 10 μM. Treatment with sodium aescinate in *Nrf2-KO* mice failed to counteract the oxidative stress induced by TBI, suggesting that also this compound exerts an activating effect on the Nrf2-ARE pathway [148].

Likewise, β-carotene exerts neuroprotective effects after TBI, through the possible involvement of the Nrf2-ARE pathway. In a TBI mouse model, administration of β-carotene (10, 20, 30, and 50 mg/kg; by gavage) improved both the cognitive and neural function. The β-carotene has increased the permeability to the BBB and protected the brain tissue from the oxidative stress TBI-induced. Indeed, especially at the dose of 30 mg/kg, the compound increased the levels of SOD and decreased those of MDA. The β-carotene after 7 days of treatment protected the neuronal cortex from TBI-induced apoptosis. Furthermore, it was shown that the treatment increased the translocation of *Nrf2* into the nucleus, decreased the expression of *Keap1*, and increased the expression of the Nrf2′s downstream effectors (NQO-1 and HO-1), both at the protein level and mRNA [149].

Another carotenoid pigment that exhibits antioxidant, anti-inflammatory, immunomodulatory, and neuroprotective properties, is astaxanthin, which has also shown promise in counteracting oxidative stress in TBI [150,151]. In this regard, the mechanisms underlying the effects of the compound were studied in a mouse model of TBI. Already one day after the injury, astaxanthin (100 mg/kg; intraperitoneal) improved the neuronal function and significantly increased the expression levels of the Nrf2 protein and mRNA, consequently also increasing that of its downstream expression (HO-1 and NQO1). Furthermore, 24 h after injury, the treatment induced an increase in *Nrf2* nuclear translocation, suggesting that the neuroprotective effects of Nrf2 against TBI-induced oxidative stress may be mediated by activation of the Nrf2/HO-1 pathway [152]. In another study, astaxanthin (25, 75, and 150 mg/kg) counteracted oxidative stress 1, 3, and 7 days after injury. Moreover, 3 days after the damage, this compound induced an improvement of the neurological functions compromised by the lesion, increasing the expression of peroxiredoxin 2 (Prx2), Nrf2, and sirtuin 1 (SIRT1). Conversely, it reduced the phosphorylation of kinase 1 for the regulation of the apoptosis signal (ASK1) and p38. Thus, it was demonstrated that astaxanthin could inhibit oxidative insults and neuronal apoptosis via activation of SIRT1/Nrf2/Prx2/ASK1/p38 signaling. Furthermore, 21 days after the damage induction, the treatment reduced neuronal apoptosis and lesion volume, suggesting that astaxanthin could improve TBI-induced neurological deficits even long term. Effects of astaxanthin on SIRT1/Nrf2/Prx2/ASK1/p38 signaling were also confirmed in vitro in H_2_O_2_-induced primary cortical neurons [153].

Tannic acid is a natural compound, recently known for reducing neurodegeneration and behavioral deficits in an ischemic stroke model [154]. For this reason, its neuromodulatory properties were investigated in a rat model of TBI. Treatment with tannic acid (50 mg/kg) was performed intraperitoneally 30 min before and 6 and 18 h after the injury. A total of 24 h after the injury, the treatment reduced neuronal damage, decreased brain edema, and improved behavioral changes. This polyphenol induced these effects by restoring GSH levels in penumbra tissue and increasing levels of antioxidant enzymes (GST, GSH peroxidase, CAT, and SOD). Additionally, the treatment also significantly increased the protein expression of PGC-1α and Nrf2, mitochondrial transcription factor A, and HO-1 after TBI. Tannic acid improves behavioral deficits, oxidative damage, and mitochondrial damage by activating the PGC-1α/Nrf2/HO-1 signaling pathway [155].

Furthermore, some isothiocyanates such as allyl isothiocyanate, extracted from radish, mustard, and wasabi, are already known for their ability to activate Nrf2 in cultured fibroblasts [156]. Treatment with allyl isothiocyanate given after an injury reduced the brain edema and BBB permeability by decreasing glial fibrillary acid protein (GFAP) and NF-κB levels, and increasing the levels of Nrf2, protein growth-associated 43 (GAP43), and neural cell adhesion molecules. This demonstrates how allyl isothiocyanate enhanced the expression of neuronal plasticity markers and endogenous antioxidant mechanisms through Nrf2 upregulation [157]. Similarly, treatment with lupeol (50 mg/kg), 7 days after the injury inhibited TBI-induced apoptotic cell death in mouse brains by reducing mitochondrial apoptotic signaling (caspase-3, Bax, cytochrome-C and Bcl2), and reduced the activation of glial cells in the cortex and hippocampus of the brain. Furthermore, lupeol promoted Nrf2/HO-1 expression and thereby reduced oxidative stress in the TBI mouse brain [158]. Furthermore, Huperzine-A, a natural sesquiterpene alkaloid, has been shown to have efficacy in improving behavioral alterations in a model of TBI [159]. In this regard, Huperzine-A (0.5 mg/kg) was administered intraperitoneally 30 min after the first injury, and once daily for one month. The treatment reduced brain edema, restored behavioral changes, and improved cognition by increasing synaptic proteins. The Huperzine-A treatment also inhibited the inflammatory response and enhanced the activity of antioxidant enzymes. Finally, this compound exerted its antioxidant effects by activating the Nrf2 pathway and promoting the translocation of Nrf2 from the cytoplasm to the nucleus [160].

Rutaecarpine derived from *Euodia rutaecarpa* can also activate the Nrf2 pathway and inhibit oxidative damage, as demonstrated in a model of craniofacial injury [161]. In a model of TBI, rutaecarpine (5, 10, 20 mg/kg) 24 h and 30 min before the lesion, and 2, 24, 48 and 72 h after TBI, improved cognitive impairment, inhibited neuronal apoptosis, and counteracted lesion-induced oxidative stress. The same result was also obtained in H_2_O_2_-induced PC12 cells. Furthermore, rutaecarpine promotes PGK1 and Nrf2 expressions, suggesting that it may exert its neuroprotective effects against TBI by activating the PGK1/Keap1/Nrf2 pathway [162]. The same result was also obtained by treatment with evodiamine (5, 10, 20 mg/kg), a quinazoline alkaloidal extracted from the fruit of *Evodia rutaecarpa* [163]. Similarly, aubucin, an iridoid glycoside, has been shown to be a promising compound due to its neuroprotective, antioxidant, and anti-inflammatory properties [164]. Its properties were tested in an in vitro model of H_2_O_2_-induced oxidative stress in primary cortical neurons. Cells were treated for 12 h with aubucin (50, 100 or 200 μg/mL). Treatment promoted the translocation of *Nrf2* from the cytoplasm to the nucleus, activating the expression of antioxidant enzymes and thereby reducing oxidative stress and reducing apoptosis. Furthermore, in vivo treatment with aubucin (20 or 40 mg/kg) administered intraperitoneally at 30 min, 12, 24, and 48 h after the injury reduced brain edema, ameliorated histological damage, cognitive deficit, and short and long-term neurologic functions. In *Nrf-KO* mice, aubucin treatment did not reverse oxidative damage, demonstrating the implication of Nrf2 underpinning the neuroprotective and antioxidant effects of this compound [165].

**Figure 5:**
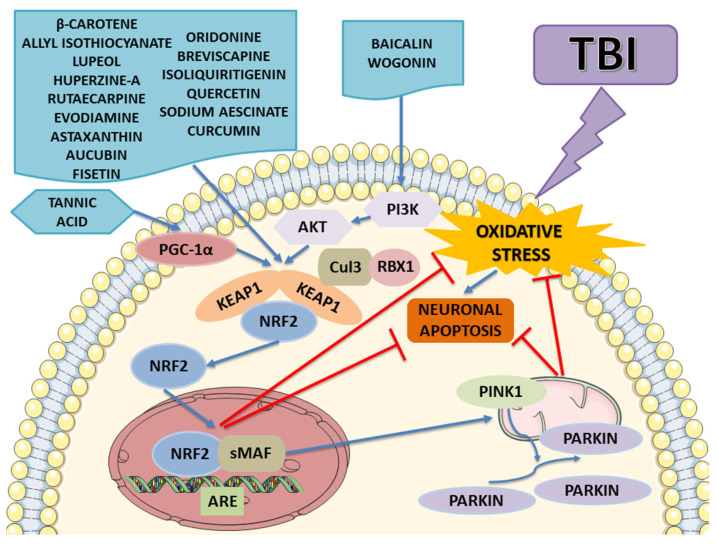
The potential molecular mechanism of natural compounds on TBI. These natural compounds can activate the expression of Nrf2 and facilitate Nrf2’s translocation from the cytosol to the nuclear, thus, inhibiting oxidative stress and protecting from neuronal apoptosis. Tannic acid positively regulates the protein expression of PGC-1α, thereby activating the Nrf2/ARE pathway. Instead, aicalin and wogonin increase the level of phosphorylated AKT and PI3K and activates Nrf2, which translocates into the nucleus to increase its downstream effectors’ production, which are responsible for anti-apoptosis and anti-oxidative effects, thus realizing the repair of TBI. The image was created using the image bank of Servier Medical Art (Available online: http://smart.servier.com/, accessed on 15 November 2022), licensed under a Creative Commons Attribution 3.0 Unported License (Available online: https://creativecommons.org/licenses/by/3.0/, accessed on 15 November 2022). Traumatic brain injury: TBI; nuclear factor E2-related factor 2: Nrf2; antioxidant response element: ARE; small muscleaponeurotic fibrosarcoma: sMAF; Kelch-like erythroid cell-derived protein with CNC homology-associated protein 1: Keap1; β-transducing repeat-containing protein: β-TrCP; Cullin 3: CUL3; RING-box protein 1: RBX1; PTEN Induced Kinase 1: PINK1; peroxisome proliferator–activated receptor gamma co-activator 1 alpha: PGC-1α; Phosphoinositide 3-kinases: PI3Ks.

The results are summarized in Table 2, which shows that most natural compounds activate the Nrf2 pathway as illustrated in Figure 5. The experimental studies have shown that the Nrf2 activation signaling pathway mediated by these compounds protects against oxidative damage mitochondrial dysfunction, apoptosis, and inflammation. The studies above-mentioned highlight that the neuroprotective potential of these compounds is linked to different mechanisms, including Nrf2/Keap1/ARE, Nrf2/PINK1/Parkin, NF-κB/TNF-α/ILs, Bax/Bcl-2/caspases, PGK1/Keap1/Nrf2, and SIRT1/Nrf2/Prx2/ASK1/p38. Thus, targeting Nrf2 signaling pathway activation is a promising therapeutic strategy for TBI.

## 8. Conclusions

The activation of Nrf2 mediated by several natural compounds promotes its translocation within the nucleus and induces the transcription of enzymes involved in counteracting oxidative stress, the mitochondrial response, and neuronal apoptosis. Nrf2 activation strategies are likely effective in preventing disease and slowing its progression. However, the current understanding of the mechanisms underlying the pathogenesis of these pathological conditions is still limited, and the different mechanisms following the activation of Nrf2/ARE remain to be further explored. Furthermore, clinical trials in patients with SCI and TBI that evaluate the therapeutic implications of these compounds are needed.

## Figures and Tables

**Table 1:** Potential natural compounds Nrf2 activators for controlling SCI symptoms and improving functional recovery.

Compound	Type of Compound	SCI Time Frame	Experimental SCI Model	Targets	Potential Effects	Type of Study	Ref.
Polydatin	A stilbenoid glucoside	Acute SCI	Rats	Nuclear Nrf2 and cytoplasmic HO-1	Polydatin is effective in ameliorating SCI, reducing oxidative stress and promoting antiapoptotic response via the Nrf2/HO-1 pathway.	In vivo	[99]
			LPS-stimulated BV2 microglia			In vitro	
Rosmarinic acid	A polyphenol	Sub-acute and chronic SCI	Rats	Nrf2/HO-1 and NF-κB	Rosmarinic acid exerts a neuroprotective effect on SCI and ameliorated the locomotor function by attenuating oxidative stress, apoptosis, and inflammation via modulating the Nrf2/HO-1 and NF-κB pathways.	In vivo	[102]
			H_2_O_2_– and LPS-induced PC12 cells			In vitro	
Ginsenoside Rb1	A saponin	Sub-acute SCI	Rats	Endothelial NOS/Nrf2/ARE	Ginsenoside Rb1 improved the hind limb function score, protected the physiological function of spinal cord tissue, and exerted a protective effect against oxidative stress injury, enhancing the activity of the antioxidant enzyme and blocking lipid peroxidation, via the eNOS/Nrf2/HO-1 pathway.	In vivo	[103]
Ginsenoside Rb1	A saponin	Acute SCI	Rats	Nrf2 and HO-1	Ginsenoside Rg1 promoted a neuroprotective effect on SCI and ameliorated motor dysfunction after an injury, exerting antioxidative and anti-inflammatory effects via regulating the Nrf2/HO-1 signaling pathway.	In vivo	[104]
Notoginsenoside R1	A saponin	Acute SCI	Rats	Nrf2 and HO-1	Notoginsenoside R1 ameliorates the SCI condition by countering oxidative stress, neuronal apoptosis, and inflammation via activating the Nrf2/HO-1 signaling pathway.	In vivo	[105]
Luteolin	A flavonoid	Acute SCI	Ischemia–reperfusion SCI rats	Nrf2	Luteolin exhibited a neuroprotective effect by alleviating oxidative stress, inhibiting inflammatory and neuronal apoptosis, probably through the signaling pathway Nrf2.	In vivo	[108]
Luteolin	A flavonoid	Acute SCI	Rats	Nrf2	The neuroprotective efficacy of luteolin depends on the suppression of oxidative stress and neuronal apoptosis through signaling pathways involving Nrf2 activation and downstream gene expression.	In vivo	[109]
Mulberrin	An oxyresveratrol glycoside	Acute SCI	Rats	Nrf2	Mulberrin could promote SCI recovery by reducing miR-337 expressions which, by regulating Nrf2, would reduce apoptosis, inflammation, and oxidative stress.	In vivo	[111]
			LPS-stimulated Astrocytes			In vitro	
Maltol	An organic compound	SCI	Rats	Nrf2/PINK1/Parkin	Maltol could stimulate mitophagy and counteract the oxidative response and neuronal cell death induced by SCI by activating the Nrf2/PINK1/Parkin pathway.	In vivo	[114]
			H_2_O_2_–-induced PC12 cells			In vitro	
Perillaldehyde	An aldehyde	Acute SCI	Ischemia–reperfusion SCI rats	Nrf2/HO-1	Perillaldehyde reduces oxidative stress and ameliorates ischemia–reperfusion SCI symptoms, probably activating the Nrf2/HO-1 signaling pathway.	In vivo	[116]
			BV2 microglia OGD/R			In vitro	
Sinomenine	An active alkaloid	Acute SCI	Rats	Nrf2	Sinomenine has the potential therapeutic efficacy agent for SCI management by inhibiting inflammation and oxidative stress via Nrf2 activation.	In vivo	[119]
			H_2_O_2_– and LPS-induced PC12 cells			In vitro	

Spinal cord injury: SCI; lipopolysaccharides: LPS; nuclear factor E2-related factor 2: Nrf2; heme oxygenase-1: HO-1; nuclear factor kappa-light chain-enhancer of activated B lymphocytes: NF-κB; nitric oxide synthase: NOS; catalase: CAT; antioxidant response element: ARE; BV2 microglia oxygen and glucose deprivation/reoxygenation: OGD/R.

**Table 2:** Potential natural compound Nrf2 activators for controlling TBI symptoms and improving functional recovery.

Compund	Type of Compound	TBI Time Frame	Experimental TBI Models	Targets	Potential Effects	Type of Study	Ref.
Oridonine	An organic compound	Acute TBI	Mice	Nrf2/HO-1 pathway	Oridonine ameliorated functional damage and neuropathological changes in animals with TBI, enhancing mitochondrial function and reducing oxidative stress-induced neuroinflammation through activating the Nrf2/HO-1 pathway.	In vivo	[133]
			H_2_O_2_-induced oxidant damage in N2a cells			In vitro	
Breviscapine	An aglycone flavonoid	Acute TBI	Rats	Nrf2/HO-1 pathway	Breviscapin treatment ameliorated TBI-induced neuron cell apoptosis and improved neurobehavioral functions through the activation of the Nrf2 pathway and its related downstream proteins (HO-1 and NQO-1).	In vivo	[135]
Isoliquiritigenin	A flavonoid	Acute TBI	Mouse TBI and *Nrf2-KO* mice	Nrf2 pathway	Isoliquiritigenin treatment attenuated lesion-induced damage by counteracting oxidative stress via Nrf2 activation, highlighting its important therapeutic potential in TBI treatment.	In vivo	[137]
			SH-SY5Y OGD/R			In vitro	
Baicalin	A major bioactive flavone	Acute TBI	Mice	Akt/Nrf2 pathway	Baicalin induces neuroprotection and prevents TBI-induced oxidative stress by activating the Akt/Nrf2 pathway.	In vivo	[139]
Wogonin	A flavonoid	Acute TBI	Mice	PI3K/Akt/Nrf2/HO-1 pathway	Wogonin protected the hippocampal damage TBI-induced by counteracting oxidative stress and neuronal death by activating the Nrf2/HO-1 pathway in a PI3K/Akt-dependent manner.	In vivo	[140]
Quercetin	A flavonoid	Acute TBI	Rats	Nrf2/HO-1 pathway	Quercetin activated the Nrf2/HO-1 pathway, thus protecting the animals from TBI-induced oxidative stress.	In vivo	[141]
Fisetin	A flavonoid	Acute TBI	Mice	Nrf2-ARE pathway	Fisetin treatment activated the Nrf2/HO-1 pathway, thus protecting the animals from TBI-induced oxidative stress and neuronal apoptosis.	In vivo	[144]
Curcumin	A diferuloylmethane	Acute TBI	Mice	Nrf2-ARE pathway	Curcumin attenuated the injury-induced oxidative stress and prevented neurological damage, possibly by activating the Nrf2-ARE pathway.	In vivo	[145]
Curcumin	A diferuloylmethane	Acute TBI	Mouse TBI and *Nrf2-KO* mice	Nrf2/HO-1 pathway	Curcumin has shown a neuroprotective role associated with the activation of the Nrf2 pathway, proving to be a potential therapeutic intervention in TBI management.	In vivo	[146]
Sodium aescinate	A triterpene saponin		Mouse TBI and *Nrf2-KO* mice	Nrf2-ARE pathway	Sodium aescinate, by activating the Nrf2-ARE pathway, exerts neuroprotective effects against oxidative stress and neuronal apoptosis TBI-induced, thus highlighting its promising therapeutic effects in the management of this pathology.	In vivo	[148]
			Neuron model of TBI			In vitro	
β-carotene	A carotenoid	Acute TBI	Mice	Nrf2/HO-1 pathway	β-carotene ameliorated brain injury after TBI by regulating the Nrf2/Keap1-mediated antioxidant pathway.	In vivo	[149]
Astaxanthin	A carotenoid pigment	Acute TBI	Mice	Nrf2/HO-1 pathway	Astaxanthin treatment promoted neuroprotective effects in the TBI mouse model probably activating the Nrf2/HO-1 signaling pathway.	In vivo	[152]
Astaxanthin	A carotenoid pigment	Acute and chronic TBI	Mouse TBI and *Nrf2-KO* mice	SIRT1/Nrf2/Prx2/ASK1/p38 signaling	Astaxanthin decreased oxidative stress and neuronal death regulating the SIRT1/Nrf2/Prx2/ASK1/p38 signaling pathway, highlighting its promising therapeutic potential in TBI even in the long term.	In vivo	[153]
			H_2_O_2_-induced oxidant damage in Primary Cortical Neurons			In vitro	
Tannic acid	A natural polyphenol	Acute TBI	Rats	PGC-1α/Nrf2/HO-1 signaling pathway	Pretreatment with tannin acid 30 min before, and 6 and 18 h after injury improved behavioral deficits, counteracting TBI-induced oxidative stress and mitochondrial damage probably by activating PGC-1α/Nrf-2/HO-1 signaling pathway.	In vivo	[155]
Allyl isothiocyanate	A organosulfur compound	Acute TBI	Mice	Nrf2 pathway	Allyl isothiocyanate treatment ameliorated TBI damage and neurological deficit, enhancing the expression of neuronal plasticity markers and reducing oxidative stress through Nrf2 upregulation.	In vivo	[157]
Lupeol	A triterpenoid	Acute TBI	Mice	Nrf2	Lupeol exerted neuroprotective effects and ameliorated memory and behavioral deficits, TBI-induced reducing glial cell activation, oxidative stress, and apoptosis likely through increasing Nrf2 levels in the brain.	In vivo	[158]
Huperzine-A	A sesquiterpene alkaloid	Acute and chronic TBI	Mice	Nrf2	Huperzine-A induces neuroprotective effects in a TBI mouse model, reducing the oxidative stress response via the Nrf2 pathway.	In vivo	[160]
Rutaecarpine	An alkaloid	Acute TBI	Mice	PGK1/Keap1/Nrf2 pathway	Rutaecarpine protected against neuronal apoptosis and oxidative stress induced by TBI, by activating the PGK1/Keap1/Nrf2 pathway.	In vivo	[162]
			H_2_O_2_-induced oxidant damage PC12			In vitro	
Evodiamine	A quinazoline alkaloidal	Acute TBI	Mice	PGK1/Keap1/Nrf2 pathway	Evodiamine protected against neuronal apoptosis and oxidative stress induced by TBI, by activating the PGK1/Keap1/Nrf2 pathway.	In vivo	[163]
			H_2_O_2_-induced PC12			In vitro	
Aucubin	An iridoid glycoside	Acute TBI	H_2_O_2_-induced oxidant damage in primary cortical neurons	Nrf2-ARE signaling pathway	Aubucin, by activating the Nrf2 pathway, attenuated TBI-induced oxidative stress and neuronal apoptosis, improving neurological outcomes, and behavioral and cognitive deficits.	In vitro	[165]
			Mouse TBI and *Nrf2-KO* mice			In vivo	

Traumatic brain injury: TBI; nuclear factor E2-related factor 2: Nrf2; heme oxygenase-1: HO-1; NADPH Quinone Dehydrogenase 1: NQO1; Nrf2-knockout: Nrf2-KO; antioxidant response elements: ARE; peroxisome proliferator–activated receptor gamma co-activator 1 alpha: PGC-1α; peroxiredoxin 2: Prx2; sirtuin 1: SIRT1; Phosphoinositide 3-kinases: PI3Ks; apoptosis signal-regulating kinase 1: ASK1; Kelch-like erythroid cell-derived protein with CNC homology-associated protein 1: Keap1; oxygen and glucose deprivation/reoxygenation: OGD/R.

## Data Availability

Not applicable.
